# Adapting a United States cancer education programme for South Africa: A participatory, culturally tailored approach using Card’s Seven-Step adaptation framework

**DOI:** 10.4102/phcfm.v18i1.5442

**Published:** 2026-07-21

**Authors:** Usangiphile Buthelezi, Buhle Lubuzo, Martha Tingen, Hlolisile Chiya, Sithabisile G. Gigaba, Bridgette Goeieman, Sibongile Ramotsela, Zamasomi Luvuno

**Affiliations:** 1 Centre for Research in Health Systems, University of KwaZulu-Natal, Durban, South Africa; 2 School of Medicine, College of Health Sciences, University of KwaZulu-Natal, Durban, South Africa; 3 Department of Population Health Sciences, Georgia Prevention Institute, Medical College of Georgia, Augusta University, Georgia, United States of America; 4 School of Nursing, College of Health Sciences, University of KwaZulu-Natal, Durban, South Africa; 5 School of Psychology, College of Humanities, University of KwaZulu-Natal, Durban, South Africa; 6 Afia Tai, Johannesburg, South Africa

**Keywords:** cancer education, programme adaptation, traditional health practitioners, faith-based leaders, cultural sensitivity, participatory action research

## Abstract

**Background:**

South Africa faces a growing cancer burden characterised by late-stage presentation, limited screening uptake and disparities in access to cancer education. While evidence-based cancer education programmes developed in high-income countries demonstrate effectiveness, direct transfer without contextual modification may compromise relevance and acceptability within resource-constrained health settings.

**Aim:**

To systematically adapt the United States (US)-based Cancer–Community Awareness Access Research & Education (c-CARE) programme as a multi-cancer education intervention for implementation within the South African primary health care (PHC) context using Card et al.’s seven-step adaptation framework.

**Setting:**

The adaptation was conducted in KwaZulu-Natal (iLembe District) and Gauteng (Johannesburg), South Africa, in collaboration with provincial Departments of Health, community health workers, traditional health practitioners and faith-based leaders.

**Methods:**

This implementation science study employed Participatory Action Research integrated with Card’s framework to guide a structured adaptation process (January 2023 – January 2024). Thirty-eight consultative meetings were conducted to review programme materials, identify linguistic, cultural and structural mismatches and implement iterative revisions while preserving core intervention components.

**Results:**

Key adaptations included the addition of cervical cancer as a priority module; incorporation of South African epidemiological data and referral pathways; translation into isiZulu and Sesotho; integration of mental health, spirituality and palliative care content; and restructuring of delivery to align with the South African Health System. Core evidence-based content, modular design and interactive pedagogical strategies were retained.

**Conclusion:**

Framework-guided adaptation enabled contextual recalibration of c-CARE while maintaining fidelity to core components.

**Contribution:**

This study provides a transparent model for adapting evidence-based cancer education interventions for culturally diverse, resource-constrained PHC systems.

## Introduction

Cancer remains a significant public health challenge in South Africa (SA), where the incidence and mortality rates reflect the country’s unique socio-economic and healthcare landscape.^1,2,3^ South Africa faces a dual burden of disease, with high rates of both communicable and non-communicable diseases, including cancer.^4,5^ Socio-economic disparities, limited healthcare infrastructure and uneven access to education exacerbate the cancer burden, particularly in rural and underserved areas.^6,7,8^ Financial constraints, long travel distances to facilities, uneven availability of screening services and shortages in diagnostic and treatment services further delay early detection and care.^9,10,11^ These challenges contribute to late-stage cancer diagnoses, which are associated with poorer prognoses and higher mortality rates.

Programmes that provide structured, culturally tailored cancer education have demonstrated improvements in knowledge, screening uptake and health-seeking behaviour in several low- and middle-income settings, particularly in relation to cervical and breast cancer prevention.^12,13^ Similarly, comprehensive education and support interventions have been associated with increased screening rates, earlier detection, improved adherence to treatment protocols and strengthened community support for patients and their families affected by cancer.^14^ These initiatives underscore the importance of community-based cancer education initiatives in equipping populations with the knowledge and resources needed to prevent, detect and manage cancer, thereby contributing to improved outcomes and reduced cancer burden in SA.^12,13^

The Georgia Cancer Center’s Cancer–Community Awareness Access Research & Education (c-CARE) programme was developed as a community-based cancer education intervention to improve prevention and early detection among medically underserved populations in the United States. Implemented across several counties in Georgia, with Augusta as a primary site, c-CARE partnered with African American healthcare providers, faith-based organisations and community health workers (CHWs) to deliver structured, cancer-specific education tailored to community needs. The programme combined facilitator guidance, participant materials, practical screening information and community engagement strategies to improve cancer awareness and encourage screening uptake. Evaluations reported meaningful improvements in cancer knowledge and screening uptake, underscoring the value of culturally responsive, community-driven education and supporting c-CARE’s selection as a suitable evidence-based programme for contextual adaptation.^15,16,17^ Although leveraging established, evidence-based programmes can enhance efficiency and increase the likelihood of successful implementation,^18,19^ direct transfer to the South African context would not be sufficient. South Africa’s socio-economic inequalities, linguistic diversity and pluralistic health-seeking practices, including the prominent role of traditional health practitioners (THPs), faith-based leaders (FBLs) and CHWs as first points of care in many communities, necessitate deliberate cultural and contextual adaptation to ensure the programme’s relevance, acceptability and educational effectiveness.^20,21,22^ Furthermore, SA’s 11 official languages linguistically present potential barriers to communication and understanding health information.^23^

In the context of these structural, linguistic, socio-economic and health system complexities, the c-CARE programme required deliberate adaptation rather than direct transfer of educational content. Therefore, the intervention needed to be restructured to ensure accessibility beyond facility-based settings and to support delivery across diverse South African communities. The adaptation process prioritised community-based delivery, engagement of locally trusted actors and alignment with existing primary health care (PHC) structures to strengthen integration and acceptability. Guided by these considerations, this study reports the systematic approach used to adapt the evidence-based c-CARE cancer education programme for the South African setting using Card et al.’s seven-step framework.^24^

## Research methods and design

### Study setting

Our study was conducted in the iLembe District Municipality in KwaZulu-Natal and Johannesburg in Gauteng province. iLembe District has a population of 678 048 and is comprised of predominantly black Africans who speak isiZulu. The district is divided into four municipalities: Mandeni, KwaDukuza, Ndwedwe and Maphumulo.^25^ It has a high human immunodeficiency virus (HIV) prevalence (43.1% among 15–49 year olds) and a cervical cancer screening rate of 61.7% in 2020, with 914 CHWs serving the area.^25,26^ Johannesburg, Africa’s leading commercial city, has nearly six million residents, with a diverse and migratory population primarily speaking isi Zulu and isi Sotho.^27^ The city also carries a substantial HIV burden, with approximately 16.4% of the people living with HIV, alongside high human papillomavirus (HPV) prevalence (85%) among women under 25 and low cervical cancer screening coverage (42.4%).^28,29,30^ At the national level, South Africa’s cervical cancer screening target aims for at least 70% coverage among eligible women under the national cancer control framework. Despite progress in some districts, screening uptake remains uneven and below optimal levels in many regions, underscoring the need for strengthened community-level education and linkage to care.

### Study design

This implementation science study employed a Participatory Action Research (PAR) design to actively engage stakeholders throughout the adaptation process.^31^ Participatory Action Research was selected because its iterative and collaborative principles align with the systematic refinement of educational interventions in response to local needs. To structure the adaptation, we applied Card et al.’s seven-step intervention adaptation framework,^24^ which provides a rigorous approach to modifying evidence-based programmes while preserving their core components. The integration of PAR and the Card framework enabled both stakeholder-driven input and structured decision-making, ensuring that modifications were contextually appropriate while maintaining fidelity to the foundational elements of the original c-CARE model.

### Programme participants

This initiative was carried out in collaboration with Augusta University in the United States (US), the South African Department of Health (DoH), traditional health leaders, FBLs and two South African-based non-profit community benefit organisations, Afia Tai and Genius Quality. Community health workers were represented by the DoH programme managers and outreach team leaders (OTLs) – professional or enrolled nurses who supervise CHWs. The collaboration with these entities ensured that the adapted c-CARE programme was relevant to the South African community, addressing its unique socio-economic conditions, cultural context and beliefs. The adapted programme was intended to strengthen community cancer education by improving knowledge, awareness, early recognition and referral among community-facing actors.

Stakeholders were purposively included to ensure representation from groups directly involved in cancer education, community engagement and PHC implementation. These included provincial DoH representatives, OTLs representing CHW programme implementation, THPs, FBLs and implementation partners from the South African and US teams. Their roles differed across the adaptation process: Department of Health representatives contributed to alignment with referral pathways, screening guidance and PHC structures; THPs and FBLs contributed to cultural, spiritual and community acceptability; and the implementation teams contributed to preserving the core components of the original c-CARE programme while adapting delivery to the South African context. Community health workers, THPs and FBLs were included because of their established community-facing roles and their potential to support cancer education, awareness, early recognition and referral within the constituencies they already serve, including households, congregations, clients and broader community networks.

### Data collection

#### Adaptation process procedures

The adaptation process was conducted over a 12-month period between January 2023 and January 2024. A total of 14 structured meetings were held between the South African and Augusta University teams (60 min each), conducted virtually via videoconference due to geographical distance. In addition, 16 consultative meetings were held in person with the provincial DoH representatives (eight per province), and eight meetings were held with traditional and FBLs across the two provinces. Stakeholders were purposively identified based on their roles in cancer care delivery, community health leadership or involvement in PHC systems (Table 1). Meeting invitations were issued via email and followed by telephonic engagement where necessary. For the purposes of this adaptation, non-healthcare professionals refer to community-based actors such as CHWs, FBLs and THPs who are not formally trained oncology providers but play influential roles in community health engagement.

**TABLE 1:** Stakeholder contribution to the cancer–Community Awareness Access Research & Education adaptation process.

Stakeholder group	Contribution to adaptation	Examples of input
Department of Health representatives	Alignment with PHC structures, screening guidance and referral pathways	Local service pathways, CHW roles and screening access points
Outreach Team Leaders or CHW Supervisors	Practical delivery considerations	Community delivery, CHW-facing language and referral feasibility
Traditional health practitioners	Cultural relevance and community acceptability	Cancer beliefs, traditional healing pathways and trust-building
Faith-based leaders	Spiritual support and community mobilisation	Spiritual care and stigma-sensitive communication
South African implementation team	Contextualisation of content and delivery	Local epidemiology, language and module restructuring
US c-CARE team	Fidelity to original programme	Retention of core educational content and modular structure

CHW, community health workers; c-CARE, cancer–Community Awareness Access Research & Education; PHC, primary health care.

Training materials were circulated electronically to stakeholders in advance of meetings, except for meetings with FBLs and THPs (due to lack of access to emails and other electronic platforms). In this article, programme materials refer to the broader c-CARE intervention package, including training manuals, facilitator guidance, educational content, teaching strategies, language, terminology, referral information and delivery arrangements. Semi-guided discussions were held using the structured pre-circulated training manuals. Sessions typically involved page-by-page review of educational content, identification of contextual mismatches and collaborative deliberation on proposed modifications. Linguistic and cultural adaptation was conducted through stakeholder review of terminology, phrasing, examples, imagery and culturally sensitive explanations of cancer and care-seeking. Formal cognitive debriefing was not conducted during this adaptation phase. However, detailed meeting notes were documented contemporaneously and used to inform subsequent revisions.

This data collection process employed a multi-faceted approach to generate comprehensive insights into the adaptation of the c-CARE programme for the South African context. It began with the retrieval and review of the original c-CARE training manuals, which covered four cancer types: multiple myeloma, lung cancer, prostate cancer and breast cancer. In response to local epidemiological needs and stakeholder input, the South African team, working collaboratively with colleagues from Augusta University, developed a fifth manual focused on cervical cancer, a priority health concern in the region. Thus, the final set of training materials comprised five manuals, four of which required contextual adaptation. Additionally, training modules on palliative care, spiritual care and mental health were developed and integrated into the programme, reflecting the expressed needs and priorities of South African stakeholders.

**FIGURE 1:**
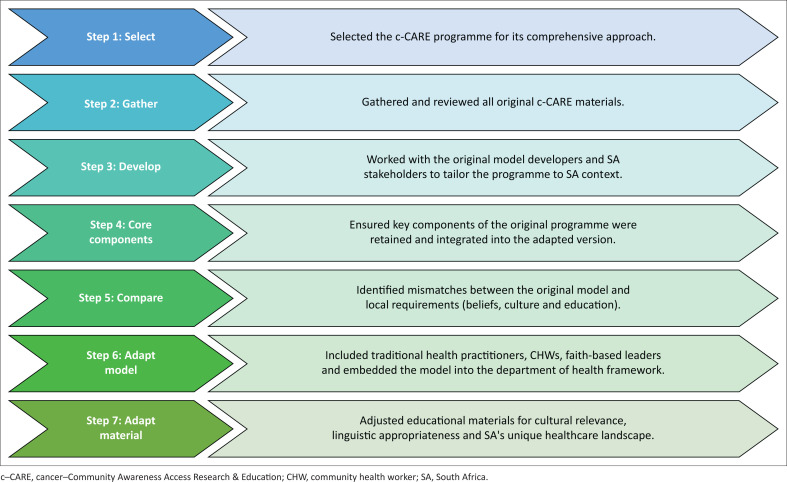
Cancer–Community Awareness Access Research & Education programme adaptation process for South Africa using card’s Seven-step framework.

In addition, the adaptation process also followed a seven-step approach by Card et al.^24^ for adopting programmes for use in new contexts^24^ (Figure 1). Card et al.’s framework provides a stepwise process for adapting effective programmes for new contexts by selecting a suitable programme, reviewing its original materials and model, identifying core components, comparing the original programme with the new context and making contextually appropriate adaptations while preserving programme fidelity. A detailed summary of the seven steps and their application in this study is provided in Online Appendix 1.

This stepwise framework encourages stakeholders to make culturally relevant changes to the programme only when necessary while adhering to the original programme’s theory of change and core components.^24^ It also emphasises the importance of aligning with the literature on best practices, ensuring that adaptations enhance the programme’s overall effectiveness and suitability for the community.

### Data analysis

Data generated through stakeholder meetings, consultative discussions and document review (training manuals) were synthesised using a framework-guided analytic approach aligned with Card et al.’s seven-step model.^24^ Detailed meeting notes were reviewed iteratively to identify recurrent themes related to cultural relevance, linguistic clarity, health system alignment and educational delivery. Suggested adaptations were categorised according to the corresponding step of the framework (e.g. identifying mismatches, defining core components, restructuring delivery). As this study reports a programme adaptation process rather than an intervention outcome evaluation, no statistical analysis was conducted; instead, the analysis was framework-guided and focused on synthesising stakeholder feedback, document review and adaptation decisions using Card et al.’s seven-step model.

Proposed modifications were assessed against two criteria: (1) preservation of core educational components identified as essential to the original c-CARE model and (2) contextual appropriateness within the South African setting. Decisions to adopt, modify or exclude suggested changes were reached through consensus between the South African and Augusta University teams. This structured approach ensured transparency in decision-making while maintaining fidelity to the foundational elements of the intervention.

Decisions to include or exclude proposed adaptations were guided by three criteria: relevance to the South African cancer burden and health system, acceptability to community-facing stakeholders and preservation of the original programme’s core educational components. Adaptations were included when they improved local relevance, clarified terminology, aligned content with South African screening or referral pathways or responded to stakeholder-identified cultural and educational needs. Suggested changes were not incorporated where they duplicated existing content, moved beyond the educational scope of the programme, required clinical services unavailable within the intended delivery context or risked changing the programme from a community cancer education intervention into a clinical training package. Final decisions were reached through consensus discussion among the South African research team and the US c-CARE team.

### Ethical considerations

Ethical clearance to conduct this study was obtained from the University of KwaZulu-Natal Biomedical Research Ethics Committee (No. BREC/00004890/2022). Written informed consent was obtained from all participants before data collection.

## Results

The results describe the structured adaptation of the c-CARE programme in accordance with Card et al.’s seven-step framework.^24^ Rather than evaluating intervention outcomes, this section reports the key modifications, stakeholder contributions and contextual refinements that emerged at each stage of the adaptation process. Guided by participatory engagement and framework-driven decision-making, each step resulted in specific adjustments to content, delivery format, educator roles and health system alignment to ensure suitability for the South African context. Figure 1 in the ‘Adaptation process procedures’ section provides an overview of the adaptation pathway, while the sections below detail the outcomes derived from each step of the framework.

### Step 1: Selecting a suitable, effective programme

Application of the first step of Card et al.’s framework involved identifying an existing evidence-based cancer education programme suitable for contextual adaptation. The c-CARE programme was selected following a review of its documented outcomes in medically underserved populations in the United States, particularly its demonstrated improvements in cancer knowledge and screening uptake.^15,16,17^

During initial consultative meetings, stakeholders, including provincial health representatives and community leaders, identified several characteristics of the original model as transferable to the South African context. These included its community-based delivery approach, structured educational curriculum and use of trusted community figures to facilitate learning. Stakeholders emphasised the relevance of training non-specialist community actors, noting similarities between the role of FBLs in Augusta and the influential role of religious and traditional leaders in South African communities. Based on this assessment, the c-CARE programme was confirmed as an appropriate foundation for structured adaptation rather than the development of a new intervention.

### Step 2: Gathering the original programme materials

The second step involved systematic retrieval and review of the original c-CARE programme materials. The Augusta University team provided the full training package, including cancer-specific manuals, facilitator guides, educational resources and implementation guidance documents. The original curriculum comprised modules addressing lung, breast, prostate and multiple myeloma cancers, alongside structured educational content covering risk factors, early detection and screening practices.

All materials were reviewed collaboratively by the South African and US teams through structured document analysis. This review aimed to identify core educational components, delivery structure, sequencing of modules and pedagogical strategies that would require preservation or contextual modification. During this process, it was noted that cervical cancer, despite being a major public health concern in SA due to high HIV prevalence and associated cervical cancer risk, was not included as a standalone module in the original curriculum. Stakeholders identified this omission as a critical contextual gap requiring inclusion in the adapted programme.

Key elements identified for retention included simplified explanations of cancer pathophysiology, structured coverage of signs and symptoms and emphasis on prevention and screening. The identification of both essential core components and contextual gaps informed subsequent steps focused on mismatch analysis and curriculum modification.

### Step 3: Developing a programme model

The third step focused on defining a localised programme model to guide delivery of the adapted c-CARE curriculum within the South African context. Stakeholder consultations identified three structural considerations requiring modification from the original model: (1) alignment with PHC and Ward-Based Outreach Team structures, (2) inclusion of culturally embedded health belief systems and (3) adaptation of language and teaching strategies to accommodate diverse literacy levels.

Representatives from provincial DoH emphasised the importance of linking educational sessions to existing referral pathways and screening services to ensure continuity between education and care. Traditional and FBLs highlighted the influence of spiritual and cultural interpretations of illness, underscoring the need to integrate culturally grounded messaging and trusted community educators into programme delivery.

Based on this input, the programme model was revised to incorporate community-based facilitators operating in coordination with local health structures rather than relying on facility-centred delivery alone. Educational materials were iteratively revised through structured review cycles between the South African and US teams, with modules circulated electronically, annotated and returned with context-specific recommendations. Revisions addressed terminology, imagery, case examples, sequencing of content and instructional pacing.

**FIGURE 2:**
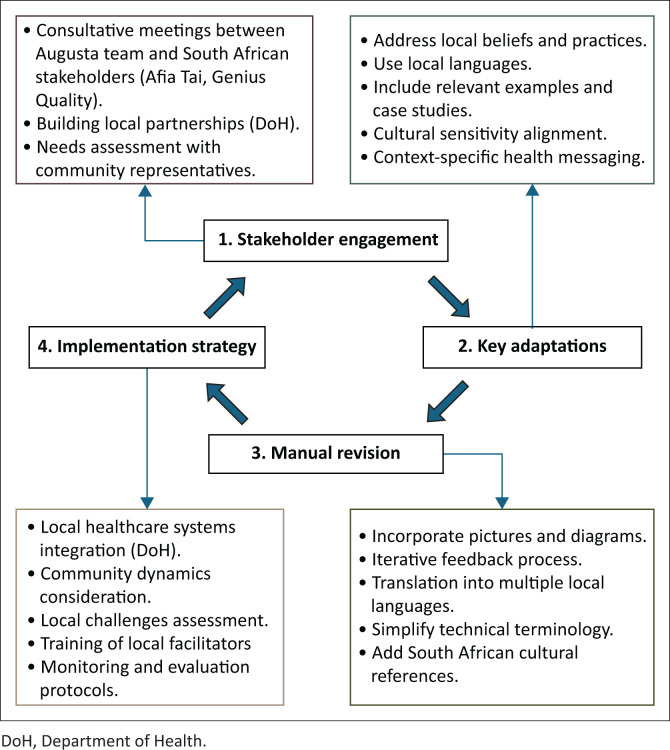
Contextualised adaptation model for cancer–Community Awareness Access Research & Education programme in South Africa.

The finalised programme model therefore reflected a hybrid structure that preserved the core educational architecture of the original intervention while redefining delivery platforms, facilitator roles and contextual framing to align with South African community and health system dynamics. Figure 2 provides a visual summary of this structured adaptation pathway.

### Step 4: Identifying the core components and best-practice characteristics

The fourth step involved identifying and preserving the core components of the original c-CARE programme to ensure fidelity during contextual modification. Through structured review, essential elements were defined as those central to the intervention’s educational architecture and delivery model. Step four was therefore used as a review point to confirm which elements of the original c-CARE programme needed to be retained before contextual adaptations were finalised.

The following components were retained in the adapted programme:

- **Evidence-based educational content:** The adapted curriculum-maintained c-CARE’s grounding in scientific literature and clinical guidelines, including structured coverage of cancer risk factors, prevention, early detection and management.
- **Comprehensive multi-cancer curriculum:** The adaptation process preserved c-CARE modular structure, addressing lung, breast, prostate, multiple myeloma and following contextual review, cervical cancer. Each module included information on signs and symptoms, modifiable risk factors and screening recommendations.
- **Community-based delivery model:** The original c-CARE programme was designed to be implemented in community settings, leveraging trusted local figures like church leaders and CHWs to deliver cancer education.^15,16,17^ In the South African context, this model was maintained and expanded to include FBLs, CHWs and THPs.
- **Interactive pedagogical strategies:** Structured workshops, guided discussions and participatory learning activities were preserved to facilitate engagement and comprehension among non-specialist audiences.
- **Structured screening guidance:** Clear explanations of screening pathways and early detection protocols were maintained and subsequently aligned with South African national health guidelines.

These core components were classified as non-negotiable elements of the intervention. Contextual adaptations implemented in subsequent steps were therefore designed to modify delivery format, language and cultural framing without altering the foundational educational structure.

### Step 5: Identifying and categorising mismatches

The fifth step involved systematic identification of contextual mismatches between the original c-CARE curriculum and the South African setting. Through collaborative review and stakeholder consultation, discrepancies were categorised into three domains: (1) linguistic and cultural mismatches, (2) content mismatches and (3) structural mismatches. These categories guided the prioritisation of targeted modifications:

- **Cultural and linguistic mismatches:** The original module, developed for an English-speaking audience in the United States, needed to be assessed for linguistic appropriateness. The diversity of languages spoken in SA, including isiZulu and other local languages, required a careful examination of terminology and phrasing to ensure clarity and comprehension. Any language, imagery, or cultural references in the original module that did not resonate with the South African community were categorised as cultural and linguistic mismatches. This included idiomatic expressions, culturally specific health beliefs and practices not applicable in SA.
- **Content mismatches:** Several elements of the original c-CARE programme were identified as mismatches to the South African context, such as references to US-specific healthcare facilities, insurance systems and treatment protocols. The use of computed tomography (CT) scan for lung cancer screening, which is less common in SA, was also flagged as irrelevant. To make the programme more locally applicable, South African-specific data on cancer incidence, mortality rates and healthcare access were incorporated, ensuring the content aligned with the realities of the local healthcare landscape.
- **Structural mismatches:** Differences in the healthcare infrastructure and socio-economic conditions between the original and target populations were categorised as structural mismatches. This included differences in healthcare access, screening and treatment services availability, as well as the overall public health environment.

### Step 6: Adapting the original programme

This step involved implementing adaptations to the c-CARE curriculum and delivery model to align with South Africa’s cultural context, linguistic diversity and socio-economic and health system realities. Adaptation decisions were informed by the core components identified in Step 4 and the contextual mismatches categorised in Step 5, as well as a contextual needs assessment that considered the national cancer burden (priority cancer types), locally relevant risk factors and barriers to screening and access to care. The following proposed changes were reviewed iteratively with key stakeholders and refined through structured feedback cycles:

#### Engagement of traditional health practitioners, faith-based leaders and community health workers

Stakeholders emphasised that cancer education in many South African communities is shaped by the influence of spiritual and cultural leaders and by patterns of medical pluralism. In response, the adapted model incorporated THPs, FBLs and CHWs as key audiences and delivery partners within the programme. These groups were positioned as locally trusted actors who frequently serve as first points of contact for community members seeking advice and support. Their inclusion informed the programme’s framing, language choices and emphasis on trust-building and culturally congruent communication.

#### Alignment with the formal local healthcare system and existing primary healthcare structures

Adaptations were implemented to align the programme with South Africa’s public sector service organisation and policy priorities. Stakeholders highlighted the importance of integrating the programme within PHC systems, including PHC re-engineering and Ward-Based Primary Health Care Outreach Teams (WBPHCOTs). In response, the curriculum was revised to incorporate locally relevant referral pathways and service navigation guidance, including information on local clinics, hospitals and available support organisations. US-specific system assumptions and references were removed or replaced with South African equivalents. These revisions ensured that educational content was linked to realistic screening and care access points within district and provincial service delivery contexts.

#### Adaptation of teaching methods and learning materials

The instructional approach was modified to align with local learning preferences, literacy variation and delivery constraints. Stakeholder feedback supported the use of visual and interactive strategies to strengthen comprehension among non-specialist audiences. Accordingly, visual aids were revised, including the addition of diagrams to improve conceptual clarity, and interactive elements were tailored to local realities. Educational examples, scenarios and supporting content were adjusted to enhance relevance and resonance in South African communities.

#### Modification of delivery format, sequencing and instructional time

The delivery format was substantially modified to reflect geographic dispersion and resource constraints affecting participation. Whereas the Augusta model delivered one 90-min module per week, the adapted curriculum was consolidated into a five-day delivery format covering all modules. In addition, approximately 3 h – 4 h were allocated to the ‘Introduction to Cancer’ module, reflecting stakeholder-identified needs related to comparatively lower baseline cancer awareness and foundational knowledge among South African participants.

To accommodate varying literacy levels and support understanding of complex medical concepts, storytelling was incorporated as a structured pedagogical strategy. Furthermore, stakeholders noted that cancer-related terminology differs across communities and villages; therefore, the adaptation process incorporated a preliminary step of identifying locally appropriate terminology and phrasing for inclusion in training delivery. This supported clearer communication while maintaining cultural and linguistic respect during programme implementation.

Collectively, these modifications operationalised the adaptation of the c-CARE programme across content, delivery format and educator engagement, while maintaining the programme’s core educational architecture.

### Step 7: Adapt the original programme materials

The final step involved consolidating all approved modifications into a finalised South African c-CARE curriculum package. The completed curriculum comprised nine structured modules: an introductory ‘Introduction to Cancer’ module; site-specific modules addressing cervical, lung, breast, prostate and multiple myeloma cancers; and dedicated modules covering mental health, spiritual care and introduction to palliative care.

The curriculum incorporated South African epidemiological data, nationally aligned screening recommendations and locally relevant referral information. Cancer-specific modules were streamlined to focus on high-priority risk factors, early detection practices and practical navigation guidance within the public health system.

Educational materials were available in English and translated versions in isiZulu and Sesotho. Visual materials reflected South African populations and healthcare settings, and simplified diagrams were incorporated to enhance clarity. The finalised training package included facilitator guides, participant manuals, structured presentation materials and supporting visual resources suitable for delivery within community-based settings. The resulting programme represented a contextually tailored version of the original c-CARE intervention while preserving its core educational structure and modular design.

## Discussion

This study documents the systematic adaptation of the c-CARE cancer education programme for the South African context using Card et al.’s seven-step framework. Rather than evaluating intervention outcomes, this paper contributes to the literature by detailing how an evidence-based cancer education model was culturally, linguistically and structurally modified to align with the realities of a lower-middle-income, pluralistic health system setting. Furthermore, this study provides a stepwise account of how adaptation decisions were identified, categorised and operationalised, an area that implementation scholars have noted remains under-documented in global health intervention transfer.^32^

A key contribution of this adaptation process was the structured identification of contextual mismatches across linguistic, content and health system domains. While much of the cultural adaptation literature emphasises translation and surface-level cultural tailoring, fewer studies explicitly differentiate between core components that require preservation and adaptable elements that can be modified without undermining programme theory.^33^ The application of Card’s framework enabled the research team to make this distinction explicit, thereby addressing the widely acknowledged fidelity–adaptation tension in implementation science.^34,35^ In contrast to ad hoc modification approaches, the structured categorisation of mismatches in this study helped prevent both superficial cultural adjustments and unintended drift from the intervention’s theoretical foundation.

The inclusion of locally trusted actors, such as THPs, FBLs and CHWs, emerged as a defining feature of the South African adaptation model. While the original c-CARE programme leveraged church-based community facilitators, the adaptation process expanded this principle to reflect South Africa’s medical pluralism and the influential role of cultural and spiritual leaders in shaping health-seeking behaviours.^36^ Although global cancer education literature acknowledges the importance of trusted community figures,^37,38^ such engagement is frequently described at a conceptual level. In the present study, stakeholder integration extended beyond endorsement to influence curriculum framing, terminology and module prioritisation. This operational embedding of community actors addresses concerns that stakeholder involvement in adaptation processes is often symbolic rather than structurally integrated.^39^

Another critical insight from the adaptation process was the need to align educational content with realistic service access pathways. Unlike the US setting, where insurance and specialised screening pathways are more readily embedded in structured systems, the South African context required integration with PHC re-engineering strategies and WBPHCOTs, including OTLs and CHWs. Furthermore, implementation research in low- and middle-income country (LMIC) settings has repeatedly shown that health education interventions risk increasing awareness without improving service utilisation when referral systems are inaccessible or poorly aligned with community messaging.^40,41^ By embedding locally feasible referral information and screening protocols into the curriculum, this adaptation sought to close that gap, ensuring that educational recommendations corresponded with actionable service options. However, this does not remove broader supply-side constraints related to affordability, geographic access, availability of screening and diagnostic services and continuity of referral pathways. This structural alignment therefore moves beyond knowledge dissemination towards system-responsive education, a dimension increasingly emphasised in non-communicable disease implementation literature.^42,43^ At the same time, the adaptation also recognises that community education alone cannot resolve health system barriers to cancer screening, diagnosis and care.

The addition of cervical cancer as a module further illustrates the importance of epidemiological contextualisation during adaptation. Literature has shown that cross-context programme transfer can inadvertently replicate the disease priorities of the source setting rather than recalibrating to local burden.^32,40^ In contrast, the inclusion of cervical cancer in the adapted curriculum reflects responsiveness to South Africa’s high HIV prevalence and associated cervical cancer risk.^44^ Similarly, the incorporation of mental health, spirituality and palliative care modules responds to stakeholder-identified priorities and reflects a broader shift in global health discourse towards integrated, people-centred approaches to non-communicable disease education.^45,46,47^ In addition, rather than simply reproducing the biomedical scope of the original intervention, the adaptation process broadened the curricular lens to reflect locally salient dimensions of illness experience.

In sum, this structured adaptation demonstrated how framework-guided modification can address several limitations identified in the literature on transferring interventions from high-income to lower-resource settings. These include inadequate documentation, superficial cultural tailoring, limited stakeholder integration and insufficient health system alignment. In addition, the iterative review process, consensus-driven modification decisions and preservation of core components illustrate how evidence-based cancer education programmes can be responsibly transferred or adapted across contexts without compromising theoretical integrity.

### Strengths and limitations

Firstly, a strength of this study lies in the transparent documentation of adaptation decisions across each step of the framework, directly addressing the under-reporting of adaptation processes frequently noted in implementation science literature. Secondly, the participatory approach enabled meaningful stakeholder engagement and grounded modifications in lived health system realities, moving beyond token consultation towards structured co-design. However, this manuscript reports the adaptation phase only. The effectiveness, feasibility and scalability of the adapted curriculum were not evaluated during this phase. This study also did not assess broader health system readiness or service capacity for the cancer types included in the adapted curriculum. While stakeholder consultation informed adaptation decisions, direct pilot testing with end-users was not conducted at this stage. Future research will focus on pilot implementation and process evaluation to assess delivery fidelity, participant engagement and integration within the community.

## Conclusion

This study demonstrates how a structured, participatory and framework-guided adaptation process can recalibrate an evidence-based cancer education programme from a high-income setting to a culturally diverse, resource-constrained health system. By systematically identifying contextual mismatches, preserving core educational components and operationalising stakeholder-informed modifications, the adapted c-CARE curriculum was reshaped to reflect South Africa’s epidemiological priorities, linguistic diversity and PHC structures. The resulting programme represents a contextually grounded, community-oriented cancer education model that maintains theoretical integrity while enhancing local relevance. This work contributes to the growing implementation science literature by providing a transparent account of cross-national intervention adaptation and establishes a foundation for subsequent pilot implementation and evaluation within South African community and PHC settings.
